# The Incorporation of Sorghum and Cowpea Protein Isolate Into Plant‐Based Burgers Improved Their Physicochemical and Sensory Properties

**DOI:** 10.1111/1750-3841.70953

**Published:** 2026-03-22

**Authors:** Andressa Alvarenga Silva, Valéria Aparecida Vieira Queiroz, Francielle Barbosa Pena, Cícero Beserra de Menezes, Mária Herminia Ferrari Felisberto, Renata Celi Lopes Toledo, Barbara Pereira da Silva, Hércia Stampini Duarte Martino

**Affiliations:** ^1^ Department of Nutrition and Health Federal University of Viçosa Viçosa Minas Gerais Brazil; ^2^ Embrapa Milho e Sorgo Sete Lagoas Minas Gerais Brazil; ^3^ Department of Food Technology Federal University of Viçosa Viçosa Minas Gerais Brazil

**Keywords:** bioactive compounds, *Sorghum bicolor* (L.) Moench, texture profile analysis, *Vigna unguiculata* (L.) Walp

## Abstract

This study aimed to evaluate the consumer acceptance test, purchase intention, and physicochemical and technological properties of cooked plant‐based burgers formulated with sorghum and cowpea protein isolate, compared with a commercial burger (CO). Five formulations were tested: F1 (tannin‐free sorghum, 19% protein), F2 (tannin‐rich sorghum, 19%), F3 (tannin‐free sorghum, 24%), F4 (tannin‐rich sorghum, 24%), and CO (commercial hamburger made from soy). No differences were observed between sample F1 and the CO regarding color preference, flavor, texture, and overall impression. Sample F2 was selected for further analysis due to its equivalent protein content and the presence of tannins in its composition, as well as exhibiting aroma, flavor, and overall impression similar to CO. Following sensory screening, only the two most accepted formulations were selected for physicochemical and technological characterization. F1 and F2 showed excellent sources of protein, dietary fiber, iron, and zinc. F2 presented the highest tannins, *p*‐coumaric and ferulic acids, 7‐methoxyapigeninidin, and antioxidant capacity, with lower pH and higher mechanical resistance. Burgers formulated with sorghum and cowpea protein isolate proved to be promising for the development of meat analogues, with the presence of anthocyanins and phenolic acids contributing to the improvement of nutritional, technological, and sensory properties.

**Practical Applications:**

Sorghum and cowpea protein isolates are sustainable alternatives for meat analogues. Tannin‐rich sorghum burgers showed higher phenolics, anthocyanins, and antioxidant capacity. Sorghum‐based burgers provided high protein, fiber, iron, and zinc contents.

## 1 Introduction

In recent years, growing consumer concerns about health, sustainability, and animal welfare have driven increased interest in plant‐based foods, particularly among vegans, vegetarians, and flexitarians. A diversified intake of legumes and grains stands out as an effective nutritional strategy for promoting healthy and sustainable diets (Aschemann‐Witzel et al. 2021; Batista et al. 2023). In this context, the global market for meat analogues is projected to grow at a compound annual growth rate of 42.2% between 2025 and 2031, driven mainly by increasing consumer preference for sustainable, plant‐based meat alternatives (Research and Markets 2025).

Meat analogues have gained prominence for offering attractive nutritional profiles, particularly in terms of protein content. However, physical and sensory characteristics such as palatability, color, texture, flavor, appearance, aroma, and juiciness still pose challenges for consumer acceptance (Andreani et al. 2023).

Sorghum (*Sorghum bicolor* L. Moench) and cowpea (*Vigna unguiculata*) have emerged as strategic raw materials for the formulation of meat analogues due to their agronomic, physicochemical, and nutritional advantages. Both crops are well adapted to low water availability and high temperature conditions, exhibiting high drought tolerance (Bi and Wang 2022; Melo et al. 2024). Additionally, sorghum is among the top five most‐produced cereals worldwide in 2024, is naturally gluten‐free, and has a lower allergenic potential compared to other cereals such as wheat, barley, and rye (FAO—Food and Agriculture Organization of the United Nations 2024; Hegde et al. 2023).

Tannin‐rich sorghum, such as the BRS 305 hybrid, contains notable bioactive compounds, including flavonoids (isorhamnetin 3‐*O*‐rutinoside and eriodictyol) and phenolic acids (caffeic and *m*‐coumaric acids) (Paes et al. 2024). In addition, cowpea protein isolate contains approximately 92% protein, representing an excellent source of plant‐based protein (Peyrano et al. 2016). Although soy protein isolate is widely used in meat analogues due to characteristics such as mild flavor and light color (Ahmad et al. 2022), its proteins are associated with allergic reactions that can range from mild to severe, including asthma and anaphylactic shock (Pi et al. 2021). In this context, cowpea protein isolate emerges as an alternative to soy, exhibiting emulsifying and gelling properties that can enhance texture and structural integrity of the final product (Horax et al. 2004). Therefore, the combination of sorghum with cowpea protein isolate represents a promising formulation strategy for plant‐based burgers.

From a technological perspective, sorghum contributes desirable properties, such as the presence of antioxidants, neutral flavor, and potential for lower production costs compared to other cereals, such as corn or rice (Khoddami et al. 2023). The interaction between proteins and carbohydrates plays a fundamental role in obtaining adequate texture, stability, and sensory properties in meat analogues (Bohrer 2019). In this context, sorghum has demonstrated favorable technological characteristics, such as neutral flavor and contribution to greater juiciness, whereas cowpea protein isolate offers emulsifying and gelling functionality, favoring the formation and stability of the structure in hamburger‐type products (Khoddami et al. 2023). Moreover, sorghum has been widely recognized as a climate‐resilient opportunity crop due to its high water‐use efficiency and tolerance to drought and elevated temperatures, in addition to its relatively low production cost and reduced demand for agricultural inputs (Mwamahonje et al. 2024), reinforcing its potential as a sustainable ingredient for plant‐based meat products.

The objective of this study was to develop and to evaluate consumer acceptance test and purchase intention of plant‐based burgers formulated with sorghum and cowpea protein isolate and to characterize the two most accepted formulations through physicochemical and technological analyses and to compare them with a commercial burger.

## 2 Materials and Methods

### 2.1 Materials

The materials used in this study included two sorghum hybrids: CMSXS 3019, a brown‐colored with high tannin content (65.5 mg CE/g; data from Embrapa Milho e Sorgo, not published), and BRS 310, a red‐colored, tannin‐free. Both were cultivated in 2023 at the experimental farm of Embrapa Milho e Sorgo, located in Sete Lagoas, Minas Gerais, Brazil (19°27′ S, 44°14′ W, 761 m altitude). Cowpea beans used for protein isolate production were purchased from a cereal distributor in the municipality of Cajuri, Minas Gerais, Brazil (20°47′ S, 42°47′ W; 648 m a.s.l.). Both sorghum hybrids were obtained from the same harvest lot, and cowpea protein isolate was produced in a single batch. All burger formulations were prepared using these same raw material batches. Burger formulations were prepared in independent production runs according to the specific analyses performed. One batch was produced exclusively for sensory evaluation and analyzed immediately after preparation. Subsequently, two additional independent batches were prepared, one for physicochemical analyses and the other for technological analyses. All burger batches were prepared using the same raw material lots and following identical processing conditions. A commercial plant‐based burger composed of textured soy protein, isolated soy protein, isolated pea protein, and vegetable fat was purchased from the same batch at retail stores in Viçosa, Minas Gerais, and used as a control. This product was selected because it represents a widely available soy‐based formulation containing protein isolate, which reflects the most common protein system used in commercial meat analogues, and because no commercial burgers combining legume‐ and cereal‐based ingredients were available at the time of purchase.

### 2.2 Preparation of Cowpea Protein Isolate and Milled Sorghum Grains

The protein isolate was prepared following the protocol described by Shevkani et al. (2015), with modifications. Cowpea grains were ground using a hammer rotor mill with a frequency inverter and temperature‐controlled chamber (model MA‐090CFT, Marconi), equipped with a 1 mm stainless steel mesh sieve to obtain the flour. A 50 g portion of this flour was dispersed in 700 mL of deionized water (1:14, w/v), and the pH was adjusted to 9 at room temperature (25°C) using a 1 mol/L NaOH solution. The mixture was homogenized for 1 h at room temperature and then centrifuged at 10,000 × *g* for 20 min (Hettich, model Rotofix 32A, Kirchlengern, Germany). The resulting supernatant had its pH adjusted to 4.5 using 1 mol/L HCl and was centrifuged again under the same conditions. The precipitated proteins were collected, frozen at −70°C, and lyophilized (CoolSafe Basic, Denmark, LaboGane) to preserve their nutritional properties.

Prior to burger preparation, the sorghum hybrids CMSXS 3019 and BRS 310 were milled using a stone mill (model Billy 2—Hawos, Bad Homburg, Germany) and subsequently sieved to obtain 2 mm particles.

### 2.3 Development of Plant‐Based Burgers

Four formulations of plant‐based burgers were developed on the basis of the minimum protein requirement of 15%, as established by Brazilian legislation (Ministry of Agriculture, Livestock and Supply/Secretariat of Agricultural Defense 2022), which regulates the standards for meat burgers. Initially, sorghum was cooked with dehydrated beetroot flour at ratios of 11.6:1 (w/w) and 10.4:1 (w/w), using water in a 1:2 (w/v) proportion. Dehydrated beetroot flour was incorporated as a natural colorant to enhance redness and standardize a meat‐like appearance among formulations, particularly considering the differences in grain color between the sorghum genotypes (CMSXS 3019, brown; BRS 310, red). The mixture was then cooked in a conventional oven (model Du Chef Plus, Safabelli, Brazil) at 180°C for 40 min. After, the burgers were prepared following the method described by Selani et al. (2016).

Each sorghum genotype (BRS 310 or CMSXS 3019) was manually mixed with cowpea protein isolate (at sorghum‐to‐isolate ratios of 3.5:1 (w/w) or 1.6:1 (w/w), respectively), dehydrated onion and garlic, smoked paprika, xanthan gum, powdered smoke, salt, and soybean oil—until a homogeneous mixture was obtained. The four formulations produced were as follows: tannin‐free sorghum burger (F1) and tannin‐sorghum burger (F2), both with 19% of protein, and tannin‐free sorghum burger (F3) and tannin‐sorghum burger (F4), both with 24% of protein. The doughs were shaped into 100 g portions using Petri dishes to standardize the format. Finally, the burgers were wrapped in plastic film and stored at −20°C until analysis, along with the commercial plant‐based burger.

### 2.4 Sensory Analysis

This study was approved by the Human Research Ethics Committee of the Federal Institute of Education, Science and Technology of Rio de Janeiro (IFRJ), under approval number 5.717.600. The sensory analysis was based on the standard consumer acceptance test methodology, which aims to capture unbiased consumer perception of sensory attributes, independent of dietary habits or prior interest in plant‐based products (Lawless and Heymann 2010; Minim 2013). Thus, 109 untrained participants (58 men and 51 women), aged between 18 and 46 years, without sorghum or cowpea protein allergies, voluntarily participated in the consumer acceptance test. Participants were recruited from the general population, including staff and students from the Federal University of Viçosa, Viçosa, MG, Brazil, and were regular consumers of burgers, regardless of dietary pattern. Prior to participation, all volunteers signed a free and informed consent form, in accordance with institutional ethical guidelines (Figure S1). The sensory evaluation was conducted under blind conditions, and participants were informed only that they were evaluating plant‐based burgers formulated with sorghum and cowpea protein isolate, without receiving any information regarding protein content, formulation differences, or the presence or absence of tannins.

Five plant‐based burger samples were evaluated by the participants, including four formulations developed with sorghum and cowpea protein isolate and one similar commercial burger. Burgers were cooked according to the method described by Selani et al. (2016), with adaptations, and cooked at 200°C for 10 min (Mondial, model AFN‐40‐BI, Brazil). After cooking, samples were cut into cubes of approximately 2 cm (20 g per formulation), placed on white trays, and coded with random three‐digit numbers. The consumer acceptance test was conducted in individual booths (Cruz et al. 2011). The formulations were served one at a time (20 g portions) after baking, with the serving order balanced among participants to minimize order and carry‐over effects, and accompanied by a response form for evaluation, as described by Macfie et al. (1989).

Consumers evaluated the degree of acceptance of the formulations based on the following attributes: overall impression, color, aroma, flavor, and texture, using a 9‐point hedonic scale, with endpoints anchored at “extremely disliked” (point 1) and “extremely liked” (point 9). Attributes with scores below 5 were classified as below average, whereas those above 5 were considered above average. Subsequently, participants indicated their purchase intent using a 5‐point scale, where 1 corresponded to “definitely would not buy” and 5 to “definitely would buy” (Villanueva et al. 2005; Meilgaard et al. 1999).

### 2.5 Physicochemical Analyses

After the consumer acceptance test, the two best rated formulations were selected on the basis of overall impression scores. Among the formulations with similar acceptance, one formulation from each sorghum genotype was selected to ensure representativeness. As tannin‐containing formulations (F2 and F4) showed similar overall impression scores, F2 was selected for further analyses due to its protein content being equivalent to that of the most accepted formulation. Proximate composition, mineral content, condensed tannins, antioxidant capacity, total anthocyanins, 3‐deoxyanthocyanidins, phenolic acids, and water‐ and oil‐holding capacities were determined in burgers dried at 55°C for 72 h, whereas color, pH, water activity (aW), technological properties, texture profile analysis (TPA), and shear force were evaluated in burgers cooked at 200°C for 10 min (Mondial, model AFN‐40‐BI, Brazil).

#### 2.5.1 Proximate Composition

The proximate composition was determined using the methodologies proposed by the Association of Official Analytical Chemistry (AOAC International 2019). Moisture content was determined by the gravimetric method, calculated as the difference between the wet and dry sample, using a forced‐air oven (72 h, 55°C; AOAC 934.01) (Nova Ética, model 400/6ND, São Paulo, Brazil). Lipid content was measured by Soxhlet extraction (AOAC 945.16) (Tecnal, model TE‐0364, Piracicaba/SP), performed under reflux for 8 h. Total ash was quantified after incineration (550°C, 6 h) in a muffle furnace (Quimis, model Q320 M, Brazil) (AOAC 923.03). Protein concentration was determined by the semimicro Kjeldahl method, using a nitrogen‐to‐protein conversion factor of 6.25 (AOAC 2001.11). Total, insoluble, and soluble dietary fiber were analyzed using the enzymatic‐gravimetric method (K‐TDFR‐200 A, Megazyme). Carbohydrate content was calculated by difference using the following equation: [100 − (% moisture + % lipids + % protein + % total dietary fiber + % ash)]. Finally, caloric value was estimated on the basis of the Atwater factors: 4 kcal/g for protein or carbohydrate and 9 kcal/g for lipids (Merrill 1995).

#### 2.5.2 Mineral Content

Mineral content in the plant‐based burger samples was determined by inductively coupled plasma atomic emission spectrometry (ICP‐AES) (Perkin Elmer, Optima 8300), using the following wavelengths: Fe—259.939 nm, Zn—206.200 nm, Mn—293.305 nm, and Cu—327.393 nm, according to the methodology described by Gomes (1996). Results were expressed in mg per 100 g of dry sample. All materials used in the analysis were previously demineralized in 10% nitric acid solution for 24 h and rinsed three times with deionized water.

#### 2.5.3 Condensed Tannins

The extract was prepared using 0.3 g of the sample in 8 mL of methanol and 1% HCl. The tubes were placed on an automatic shaker for 20 min (model BHB 800, Benfer, Brazil) to allow extraction. Subsequently, the solution was centrifuged for 10 min at 3000*g* (model NF1200R, Nüve, Ankara, Turkey). The supernatant was transferred to another tube and stored under refrigeration until analysis. Condensed tannins were determined by the vanillin–HCl method (Price et al. 1978). Absorbance was measured at 500 nm using a spectrophotometer (Varioskan Lux, Thermo Scientific, USA). Results were expressed as milligrams of catechin equivalent per gram of sample, based on a catechin calibration curve.

#### 2.5.4 Antioxidant Capacity

The antioxidant capacity was determined by Trolox equivalent antioxidant capacity (TEAC) assay. One gram of dried and defatted flour from each plant‐based burger was extracted with 20 mL of a solvent mixture containing methanol and acetone (50:70, v/v), according to Rufino et al. (2007). Samples were agitated in an automatic shaker for 16 h and centrifuged at 3000 × *g* for 10 min (model NF1200R, Nüve, Ankara, Turkey) both at room temperature. The supernatant was collected and stored under refrigeration until analysis (Awika and Rooney 2004). The TEAC assay was performed according to Leite‐Legatti et al. (2012) and Pavan et al. (2014), with minor modifications. The ABTS•+ radical cation (2,2′‐azinobis‐(3‐ethylbenzothiazoline‐6‐sulfonic acid)) was generated by mixing 88 µL potassium persulfate (140 mmol/L) with 5 mL ABTS solution (7 mmol/L), followed by incubation in the dark at room temperature for 16 h. The working solution was diluted in ethanol (95%, v/v) to an absorbance of 0.70 ± 0.02 at 734 nm, measured using a spectrophotometer (Varioskan Lux, Thermo Scientific, USA). For the assay, 3 mL of the ABTS•+ solution was mixed with 30 µL of extract. After incubation in the dark for 6 min, absorbance was measured at 734 nm using a spectrophotometer (Varioskan Lux, ThermoFisher Scientific, USA). Antioxidant capacity was expressed as µmol trolox/g, based on a Trolox calibration curve (*y* = 0.6395*x* + 0.3452; *R* ^2^ = 0.9993).

#### 2.5.5 Total Anthocyanin and 3‐Desoxyanthocyanidins

The quantification of total anthocyanins, including the 3‐desoxyanthocyanidins (luteolinidin, apigeninidin, 5‐methoxyluteolinidin, and 7‐methoxyapigeninidin), was performed using high‐performance liquid chromatography (HPLC), according to the method described by Yang et al. (2009, 2012), with adaptations. Extraction was carried out with 20 mL of methanol acidified with 1% HCl (v/v) per gram of sample (w/v), under agitation at 200 rpm for 2 h (IKA, model Lab Dancer, Baden‐Württemberg, Germany), followed by centrifugation at 3000 rpm for 10 min (Hettich, model Rotofix 32A, Kirchlengern, Germany). The extracts were filtered through 0.22 µm membranes and analyzed using an HPLC system equipped with a C18 column, diode‐array detector set at 480 nm, and column temperature maintained at 35°C (Waters, model Alliance 2695, Milford, MA, USA). The mobile phase consisted of water with 4% of formic acid and HPLC‐grade acetonitrile, using a gradient elution program as described by Yang et al. (2009). Compound identification was based on retention times and UV–visible absorption spectra of the standards, whereas quantification was performed using calibration curves with coefficients of determination (*R* ^2^) greater than 0.99.

#### 2.5.6 Phenolic Acid

Free and bound phenolic acids were extracted and quantified according to the methodologies proposed by Shao et al. (2014) and Chiremba et al. (2012), with modifications. For free phenolic acids, 0.5 g of dry sample was extracted with 10 mL of methanol:water (80:20, v/v) under orbital agitation for 2 h at room temperature (IKA, model Lab Dancer, Baden‐Württemberg, Germany). Extracts were centrifuged at 3000 rpm for 10 min (Hettich, model Rotofix 32A, Kirchlengern, Germany), and the supernatant was evaporated under reduced pressure at 50°C using a rotary evaporator. The residue was resuspended in 1 mL of methanol:water (1:1, v/v), vortexed for 60 s, filtered through a 0.22 µm PTFE membrane, and analyzed by HPLC (Waters, model Alliance 2695, Milford, MA, USA).

For bound phenolic acids, the solid residue remaining after free phenolic extraction was hydrolyzed with 10 mL of 2 M NaOH under magnetic stirring for 2 h at room temperature. The hydrolysate was acidified with concentrated HCl, followed by liquid–liquid extraction with ethyl acetate (3 × 30 mL). The combined organic phases were evaporated at 50°C, resuspended in 4 mL of methanol:water (1:1, v/v), filtered (0.22 µm), and subjected to HPLC analysis.

Chromatographic analyses were performed on an HPLC system equipped with a reverse‐phase C18 column (250 × 4.6 mm^2^, 5 µm particle size), maintained at 38°C (Waters, model Alliance 2695, Milford, MA, USA). The mobile phase consisted of water containing 1% (v/v) acetic acid (solvent A) and acetonitrile (solvent B), operated in gradient mode at a flow rate of 1.0 mL/min. The injection volume was 20 µL, and detection was carried out at 320 nm. Phenolic acids were identified by comparison of retention times and UV spectra with authentic external standards (caffeic, *p*‐coumaric, ferulic, and sinapic acids). Quantification was performed using external calibration curves prepared at five concentration levels (10–100 µg/mL; *R* ^2^ > 0.99), and results were expressed as µg/g of dry sample.

#### 2.5.7 Water‐Holding Capacity (WHC) and Oil‐Holding Capacity (OHC)

The WHC and OHC of the plant‐based burgers were determined according to Stone et al. (2015), with minor modifications. Approximately 0.05 g of dried sample was mixed with 0.5 g of distilled water for WHC or soybean oil for OHC (1:10, w/w) in microtubes. Samples were vortexed for 10 s using a vortex mixer (Phoenix, Recnal, Brazil), with additional vortexing every 5 min for a total of 30 min. The tubes were then centrifuged at 10,000 × *g* for 15 min using a centrifuge (Fanem, model 204, São Paulo, Brazil). After centrifugation, the supernatants were carefully discarded, and the pellets were weighed to calculate WHC and OHC.

#### 2.5.8 Color

The color parameters of the plant‐based burgers were evaluated using a Color Reader CR‐10 colorimeter (Konica Minolta, Tokyo, Japan), based on the CIE Lab scale. The device directly provided the values of L* (lightness, ranging from 0 black to 100 white), a* (ranging from 128 for green to +127 for red), and b* (from 128 for blue to +127 for yellow). On the basis of the a* and b* values, chroma (C*), which expresses color saturation, with higher values indicating more vivid colors and lower values indicating duller colors, and hue angle (*h*°), which represents the predominant hue and ranges from 0° to 360°, depending on its position on the color wheel, were also calculated.

#### 2.5.9 pH and aW

For pH analysis, 5 g of cooked sample was ground and transferred to a beaker, to which 50 mL of distilled water (1:10, w/v) was added, according to Hollweg et al. (2024). The pH of the solution was measured at room temperature (25°C) using a pH meter (model P200, Bel Engineering s.r.l., Monza, Italy), previously calibrated with buffer solutions at pH 4.0, pH 7.0, and pH 10. aW was determined in cooked samples, in the consumption form, using an AquaLab Series 4 TEV analyzer (Decagon Devices, Inc., Pullman, WA, USA).

#### 2.5.10 Technological Properties

2.5.10.1

Cooking Loss. Cooking weight loss of the plant‐based burger samples was determined according to the method of Samard et al. (2021), by analyzing the weight difference between raw and cooked burger samples. Three measurements were taken for each burger, and the mean value was calculated. The percentage of cooking loss was determined using the following formula: [cooking loss (%) = (raw patty weight (g) − cooked patty weight (g))/100] × 100.

2.5.10.2

Moisture Retention. Moisture retention was measured and calculated following the methodology described by Samard et al. (2021): moisture retention (%) = (cooking yield (%) × moisture of cooked patty (g))/100 and cooking yield (%) = (cooked patty weight (g)/raw patty weight) × 100.

2.5.10.3

Diameter Reduction. Burger diameters were calculated according to the method described by Samard et al. (2021) based on the difference between the diameters before and after baking.

### 2.6 Texture Profile Analysis

TPA was performed using a TA‐XT2i texture analyzer (Stable Micro Systems Ltd., Godalming, Surrey, UK) equipped with a 5 kg load cell and a 15 mm cylindrical compression probe (P/15). Samples were analyzed in triplicate, using square‐shaped portions (2 cm × 2 cm) to ensure test accuracy.

Compression was set to 20% deformation, and tests were carried out at a speed of 5 mm/s with a force of 0.0490 N. The evaluated parameters included hardness, chewiness, gumminess, and cohesiveness.

### 2.7 Shear Force Analysis

Shear force was determined using a TA‐XT2i texture analyzer (Stable Micro Systems Ltd., Godalming, Surrey, UK) equipped with a 5 kg load cell and a guillotine‐type blade probe. Samples were evaluated in sextuplicate using square‐shaped portions (2 cm × 2 cm), which were cut to 95% of their height at a cutting speed of 1 mm/s.

### 2.8 Statistical Analysis

Sensory acceptance test data were interpreted through the construction of Internal Preference Maps using SensoMaker software (version v1.94, Federal University of Lavras, Lavras, MG, Brazil).

To compare the mean values obtained in the sensory acceptance test, physicochemical analyses, texture profile, and shear force, the data were initially subjected to the Kolmogorov–Smirnov and Shapiro–Wilk tests to assess normality. After confirming the normal distribution, a one‐way analysis of variance (ANOVA) was performed, followed by Tukey's multiple comparisons test, adopting a significance level of 5% (*p* < 0.05). Purchase intention data were analyzed using the nonparametric Kruskal–Wallis test, followed by Dunn's multiple comparisons test. To evaluate the correlation between instrumental and sensory variables, Pearson correlation was used, with *p* < 0.05 considered statistically significant. Statistical analyses were performed using GraphPad Prism software (version 8.0, Dotmatics, Boston, Massachusetts, USA).

## 3 Results

### 3.1 Consumer Acceptance Test

**FIGURE 1:**
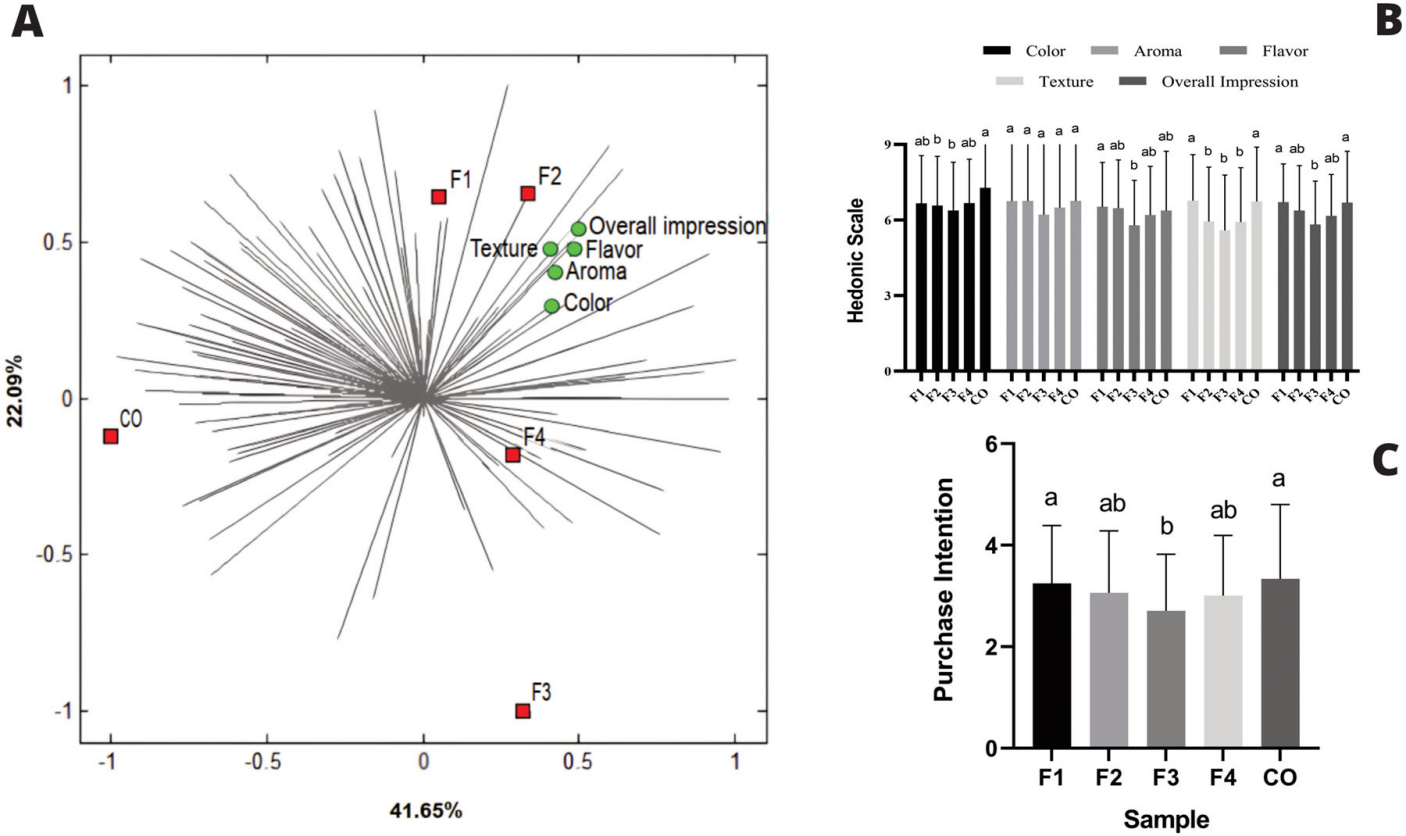
Consumer acceptance test and purchase intention of plant‐based burger formulations. (A) Internal preference map of the plant‐based burger samples based on consumers’ sensory impressions; (B) mean hedonic scale scores for color, aroma, flavor, texture, and overall impression; (C) mean purchase intention scores. F1: burger with tannin‐free sorghum and 19% protein; F2: burger with tannin‐sorghum and 19% protein; F3: burger with tannin‐free sorghum and 24% protein; F4: burger with tannin‐sorghum and 24% protein; CO: commercial plant‐based burger. Identical letters indicate no significant difference (*p* > 0.05) according to Tukey's test.

According to the Internal Preference Mapping (Figure 1A), the formulations F1 and F2, prepared with tannin‐free and tannin‐sorghum, respectively, both with 19% of protein, were positioned near the vectors for the sensory attributes “overall impression,” “flavor,” “aroma,” and “color,” indicating higher scores for these sensory characteristics. However, the distribution of the preference vectors showed that these formulations were not broadly preferred by consumers. In contrast, the formulation F3, produced with tannin‐free sorghum and 24% of protein, was located in an opposite quadrant from the sensory attributes, indicating a lower association with the consumer acceptance test characteristics. Formulation F4, prepared with tannin‐sorghum and 24% of protein, was positioned near the center of the preference map, in a region more distant and with low density of the main sensory vectors, suggesting a more neutral consumer response, with no strong association with any particular sensory attributes. The commercial burger (CO) showed the highest number of preference vectors pointing toward its position, indicating high overall acceptance. However, its location in the opposite quadrant relative to the sensory attributes suggests that this acceptance was not strongly associated with any specific sensory descriptor evaluated in this study.

There were no significant differences among the formulations for the aroma attribute. Samples F1 and CO showed no significant differences in color, flavor, texture, and overall impression. However, the formulation F2 performed worse than F1 and CO in terms of color and texture. Formulations F3 and F4 showed the lowest average scores for most sensory attributes, consistent with their peripheral positions on the internal preference map (Figure 1A,B).

Regarding purchase intent, the formulations F1 and CO presented similar and highest average scores, with values close to 5, corresponding to the category “definitely would buy.” Despite being aligned with favorable sensory attributes in the preference map, sample F2 had the lowest mean score and differed significantly from formulation F1 (Figure 1C).

On the basis of the sensory results, formulations F1 and F2 that correspond, respectively, to sorghum burgers with tannin‐free sorghum and with tannin‐sorghum and 19% of protein were selected for subsequent physicochemical analyses, considering their alignment with the most valued sensory attributes and their relative performance in the internal preference map and quantitative sensory attributes.

### 3.2 Chemical Analyses

Regarding macronutrients (Table 1), formulation F2 showed the highest carbohydrate content (40.80 ± 0.17 g/100 g), followed by F1 (39.40 ± 0.29 g/100 g), whereas the commercial sample (CO) presented the lowest value (9.02 ± 0.24 g/100 g), significantly differing from the developed formulations. Regarding protein content, F1 and F2 (22.40 ± 0.44 and 20.30 ± 0.18 g/100 g, respectively) did not differ significantly from each other. On the other hand, for lipid content, both formulations showed lower values than CO, which stood out for its high lipid content (30.90 ± 0.30 g/100 g). No significant differences were observed among F1, F2, and CO for total dietary fiber and its fractions (soluble and insoluble).

**TABLE 1:** Chemical composition of ready‐to‐eat plant‐based burger formulations on a dry matter.

	Characterization of plant‐based burger		
Compounds	F1	F2	CO
Proximate composition (g/100 g)			
Carbohydrate	39.40 ± 0.29^b^	40.80 ± 0.17^a^	9.02 ± 0.24^c^
Protein	19.40 ± 0.08^b^	19.37 ± 0.05^b^	37.57 ± 0.11^a^
Total dietary fiber	13.38 ± 0.64^a^	14.09 ± 0.17^a^	15.20 ± 0.57^a^
Soluble fiber	1.79 ± 0.89^a^	1.39 ± 0.17^a^	2.30 ± 0.17^a^
Insoluble fiber	11.59 ± 1.53^a^	12.70 ± 0.34^a^	12.90 ± 0.39^a^
Moisture	48.22 ± 0.11^b^	48.72 ± 0.18^b^	66.73 ± 0.22^a^
Lipids	22.40 ± 0.44^b^	20.30 ± 0.18^c^	30.90 ± 0.30^a^
Ash	5.42 ± 0.04^b^	5.44 ± 0.06^b^	7.31 ± 0.01^a^
**Total caloric value (kcal/100 g)**	436.80 ± 4.15^b^	423.38 ± 2.10^c^	464.46 ± 2.13^a^
Cu (mg/100 g)	0.29 ± 0.06^b^	0.28 ± 0.11^b^	0.46 ± 0.28^a^
Fe (mg/100 g)	9.66 ± 2.49^a^	9.18 ± 0.21^a^	7.18 ± 0.89^b^
Zn (mg/100 g)	2.21 ± 0.14^b^	2.29 ± 0.03^b^	2.66 ± 0.19^a^
Mn (mg/100 g)	1.84 ± 0.60^b^	1.09 ± 0.35^c^	2.35 ± 0.41^a^
Condensed tannin (mg EC/g)	ND	6.96 ± 0.03	ND
Antioxidant capacity (µmol trolox/g)	3.44 ± 0.08^c^	5.16 ± 0.06^a^	4.41 ± 0.20^b^

*Note*: F1: burger with tannin‐free sorghum and 19% of protein; F2: burger with tannin‐sorghum and 19% of protein; CO: commercial plant‐based burger. Identical letters in line indicate no significant difference (*p* > 0.05) according to Tukey's test.

Regarding ash contents (Table 1), formulations F1 and F2 presented the lowest values, whereas CO showed higher values. In the mineral analysis, F1 and F2 showed higher iron (Fe) concentrations (9.66 ± 2.49 and 9.18 ± 0.21 mg/100 g, respectively) compared to CO (7.18 ± 0.89 mg/100 g). For copper (Cu) and zinc (Zn), F1 and F2 showed lower contents than CO, with values of approximately 0.30 mg/100 g of Cu and 2.20 mg/100 g of Zn, whereas CO presented 0.46 mg/100 g of Cu and 2.66 mg/100 g of Zn. For manganese (Mn), the formulation F2 had the lowest content (1.09 ± 0.35 mg/100 g) compared to the other samples.

As expected, the condensed tannins (Table 1) were found only in the F2 (6.96 ± 0.03 mg EC/g).

In terms of antioxidant capacity (Table 1), the formulation F2 showed the highest score (5.16 ± 0.06 µmol trolox/g), followed by CO (4.41 ± 0.20 µmol trolox/g), whereas F1 exhibited the lowest antioxidant potential (3.44 ± 0.08 µmol trolox/g).

**FIGURE 2:**
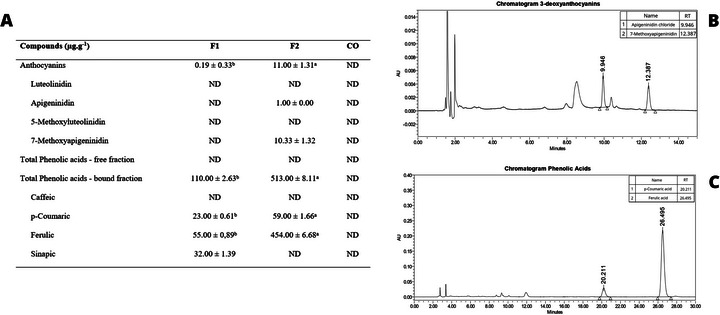
Anthocyanin and phenolic compound profile in plant‐based burger formulations. (A) Quantification of anthocyanins and phenolic acids in formulations F1, F2, and CO. (B) Representative chromatogram of the anthocyanins identified in the formulation F2. (C) Representative chromatogram of the phenolic acids present in the formulation F2. F1: burger with tannin‐free sorghum and 19% of protein; F2: burger with tannin‐sorghum and 19% of protein; CO: commercial plant‐based burger. Identical letters indicate no significant difference (*p* > 0.05) according to Tukey's test. RT, retention time.

Formulation F2, prepared with tannin‐sorghum, showed higher concentrations of anthocyanins (11.00 ± 1.31 µg/g) and phenolic compounds in the bound fraction (513.00 ± 8.11 µg/g) compared to the other samples (Figure 2). Among the bound phenolic compounds, ferulic acid (454.00 ± 6.68 µg/g) and *p*‐coumaric acid (59.00 ± 1.66 µg/g) were the most prominent. In contrast, formulation F1, prepared with tannin‐free sorghum, presented lower levels of these compounds, whereas no phenolic compounds or anthocyanins were detected in the commercial sample (CO).

In the chromatogram, for formulation F2, the apigeninidin chloride and 7‐methoxy apigeninidin were the two major peaks identified in the anthocyanins (Figure 2B). To phenolic acids, the *p*‐coumaric and ferulic acids were the two major peaks (Figure 2C).

### 3.3 Physical Analyses

**FIGURE 3:**
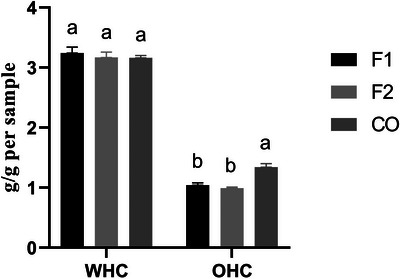
Water and oil‐holding capacity of plant‐based burgers. F1: tannin‐free sorghum burger (19% of protein); F2: tannin‐sorghum burger (19% of protein); CO: commercial plant‐based burger. Bars represent mean ± SD (*n* = 3). Different letters indicate significant differences among samples (*p* < 0.05, Tukey's test).

No significant differences were observed among formulations for WHC. In contrast, OHC differed, with F1 and F2 (*p* < 0.05) showing lower values than CO (Figure 3).

The evaluated color parameters showed that lightness (L*), which represents sample brightness, was lower in formulations F1 and F2 (33.53 ± 0.85 and 32.84 ± 1.45, respectively), indicating that they were the darkest samples. The commercial burger (CO) exhibited higher L* values (39.42 ± 1.40), suggesting a lighter appearance. The a* parameter reflects the red–green spectrum; F1 had the highest a* value (16.67 ± 1.25), indicating a redder hue, followed by F2 (14.64 ± 0.62) and CO (13.73 ± 1.34), all significantly different from one another. Regarding the b* parameter, which indicates the transition between blue and yellow tones, CO showed the highest value (14.83 ± 1.16), suggesting a more yellowish color, followed by F1 (9.62 ± 0.58). F2 had the lowest b* value (7.97 ± 0.74), suggesting less yellow contribution.

For the chroma index (C*), which indicates color saturation, F1 and F2 had lower values (19.25 ± 1.33 and 15.88 ± 1.52, respectively) than CO (20.85 ± 1.19), reflecting lower color saturation. Lastly, regarding hue angle (*h*°), which describes the dominant color tone, formulations F1 and F2 did not differ significantly from each other, whereas CO had a higher value (45.29 ± 1.49), indicating a tendency toward more yellowish hues (Table 2).

**TABLE 2:** Color parameters (L*, a*, b*, C*, and *h*°) of plant‐based cooked burger formulations.

Sample	L*	a*	b*	C*	*h*°	Raw burger	Cooked burger
F1	33.53 ± 0.85^b^	16.67 ± 1.25^a^	9.62 ± 0.58^b^	19.25 ± 1.33^b^	30.03 ± 1.01^b^	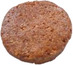	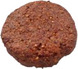
F2	32.84 ± 1.45^b^	14.64 ± 0.62^b^	7.97 ± 0.74^c^	15.88 ± 1.52^c^	30.21 ± 1.02^b^	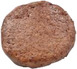	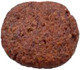
CO	39.42 ± 1.40^a^	13.73 ± 1.34^c^	14.83 ± 1.16^a^	20.85 ± 1.19^a^	45.29 ± 1.49^a^	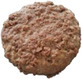	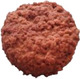

*Note*: F1: burger with tannin‐free sorghum and 19% of protein; F2: burger with tannin‐sorghum and 19% of protein; CO: commercial plant‐based burger. Identical letters in line indicate no significant difference (*p* > 0.05) according to Tukey's test.

The pH values of formulations F1 and F2 did not differ significantly from each other (5.09 ± 0.31 and 4.63 ± 0.14, respectively) but were lower than that of the commercial sample (CO), which showed the highest pH (5.96 ± 0.02). Regarding aW, a similar trend was observed for F1 and F2 (0.96 ± 0.01 and 0.97 ± 0.02), both lower than CO (0.98 ± 0.00).

For cooking yield, formulation F1 (92.54% ± 0.64%) had a higher value than F2 (90.22% ± 0.86%) and was comparable to CO (93.78% ± 0.64%). Consequently, cooking loss was lower for F1 (7.46% ± 0.64%) and CO (6.22% ± 0.57%). Moisture retention was similar between F1 and F2 (44.63 ± 0.31 and 43.96 ± 0.42), but both were significantly lower than CO (62.58 ± 0.38), indicating a reduced ability to retain water after cooking. Finally, no significant difference was observed among the formulations in terms of diameter reduction (Table 3).

**TABLE 3:** Technological properties of plant‐based burger formulations.

Properties	F1	F2	CO
pH	5.09 ± 0.31^b^	4.63 ± 0.14^b^	5.96 ± 0.02^a^
aW	0.96 ± 0.01^b^	0.97 ± 0.02^b^	0.98 ± 0.00^a^
Cooking yield (%)	92.54 ± 0.64^a^	90.22 ± 0.86^b^	93.78 ± 0.64^a^
Cooking loss (%)	7.46 ± 0.64^b^	9.78 ± 0.86^a^	6.22 ± 0.57^b^
Moisture retention (%)	44.63 ± 0.31^b^	43.96 ± 0.42^b^	62.58 ± 0.38^a^
Diameter change (%)	5.51 ± 0.65^a^	5.88 ± 0.00^a^	4.81 ± 1.95^a^

*Note*: F1: burger with tannin‐free sorghum and 19% of protein; F2: burger with tannin‐sorghum with 19% of protein; CO: commercial plant‐based burger. Identical letters in line indicate no significant difference (*p* > 0.05) according to Tukey's test.

In the TPA (Table 4), formulation F2 showed the highest hardness (28.75 ± 5.90 N) compared to the other formulations, followed by F1 (19.01 ± 4.91 N), both of which were significantly harder than the commercial sample (CO) (11.79 ± 2.01 N).

**TABLE 4:** Texture profile analysis and shear force evaluation of plant‐based burgers.

Textural properties	F1	F2	CO
Hardness (N)	19.01 ± 4.91^b^	28.75 ± 5.90^a^	11.79 ± 2.01^c^
Chewiness	132.93 ± 48.69^b^	187.46 ± 83.69^b^	628.86 ± 76.77^a^
Gumminess	473.34 ± 125.29^b^	648.59 ± 208.50^ab^	813.98 ± 91.04^a^
Cohesiveness	0.23 ± 0.03^b^	0.23 ± 0.02^b^	0.68 ± 0.04^a^
Force *X* (N)	4.28 ± 1.04^ab^	5.58 ± 0.99^a^	4.23 ± 0.33^b^
Peak force (N)	1.11 ± 0.58^ab^	2.79 ± 2.04^a^	0.58 ± 0.10^b^

*Note*: F1: burger with tannin‐free sorghum and 19% of protein; F2: burger with tannin‐sorghum and 19% of protein; CO: commercial plant‐based burger. Identical letters in line indicate no significant difference (*p* > 0.05) according to Tukey's test.

Regarding chewiness, F1 and F2 did not differ significantly from each other (132.93 ± 48.69 and 187.46 ± 83.69, respectively) but were both significantly lower than CO (628.86 ± 76.77), which presented a value approximately three times higher, indicating greater effort required during mastication.

For gumminess and cohesiveness, formulation F2 (648.59 ± 208.50) did not differ significantly from F1 and CO (0.68 ± 0.04). However, F1 (473.34 ± 125.29) was statistically different from CO (813.98 ± 91.04).

For the force *X* parameter, formulation F1 (4.28 ± 1.04 N) did not show a significant difference compared to the other formulations (F2 and CO). However, F2 (2.79 ± 2.04 N) was higher than CO (4.23 ± 0.33 N). Similarly, the peak force parameter, which represents the initial effort required to break the sample, followed a similar trend to force *X*. Formulation F2 (2.79 ± 2.04 N) was significantly higher than CO (0.58 ± 0.10 N), whereas F1 (1.11 ± 0.58 N) did not differ statistically from either (Table 4).

In the correlation analysis, hardness showed a direct association with maximum force, force *X*, anthocyanin content, and bound phenolic acids. Conversely, hardness was inversely correlated with sensory texture perception, cohesiveness, chewiness, and gumminess. Chewiness, in turn, was positively correlated with gumminess and cohesiveness, while showing an inverse relationship with hardness and maximum force.

**FIGURE 4:**
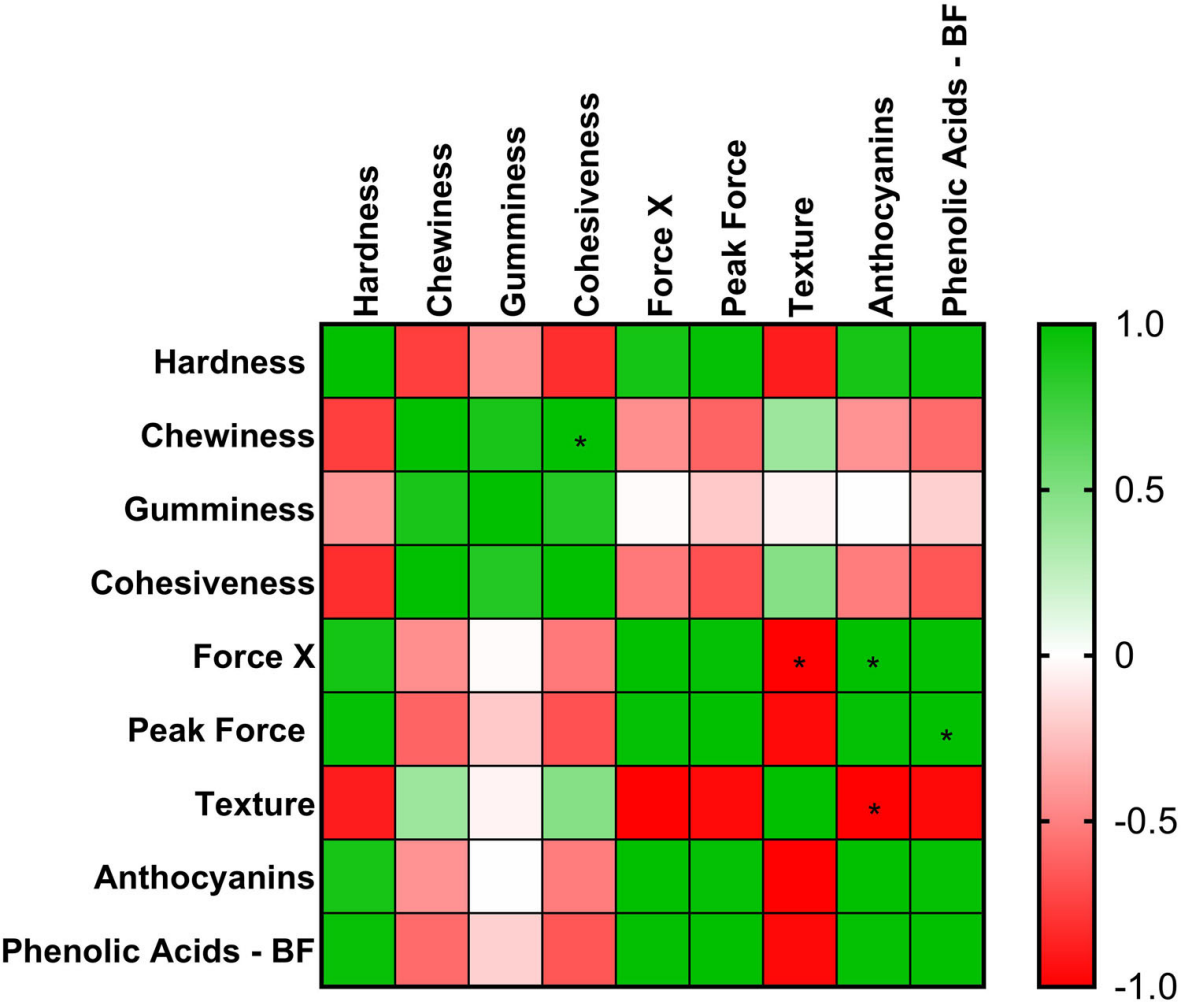
Pearson correlation coefficient between sensory texture parameters, instrumental texture profile analysis (TPA), peak force, force *X*, anthocyanins, and phenolic acids in plant‐based burgers. The color scale represents the strength and direction of the correlations: green indicates positive/direct correlations, red indicates negative/inverse correlations, and white indicates no correlation. (*) indicates a statistically significant difference at *p* < 0.05. BF, bound fraction.

Maximum force was directly correlated with hardness, force *X*, anthocyanins, and bound phenolic acids and inversely associated with the sensory perception of texture. Similarly, phenolic compounds (anthocyanins and bound phenolic acids) were positively correlated with hardness and maximum force and negatively correlated with sensory texture perception (Figure 4).

## 4 Discussion

The development of plant‐based meat analogues currently represents one of the main challenges and opportunities in the food industry. Innovating in formulations that combine desirable sensory attributes, technological quality, and nutritional value is essential to ensure the competitiveness of these products compared to animal‐based alternatives. In this context, the present study aimed to contribute to addressing these challenges through the development and physiochemical and sensory characterization of plant‐based burgers prepared with different sorghum hybrids and cowpea protein isolate.

Regarding sensory attributes, the formulations with tannin‐free sorghum (F1) and with tannin‐sorghum (F2), both containing 19% protein, were more closely associated with overall impression, flavor, aroma, and color, indicating higher consumer acceptance compared with formulations F3 and F4 (24% protein). Despite performing well in sensory attributes, F2 received the lowest mean score for purchase intention, in contrast to F1 and the commercial sample (CO), which achieved the best results and were more positively received in this aspect. This superior performance of F1 and F2 may be related to the lower concentration of cowpea protein isolate in these formulations compared to the tannin‐free (F3) and tannin‐containing (F4) burgers with 24% of protein. The high protein content of cowpea protein isolate (85.9% to 87.7%) has been associated with increased hardness and chewiness in meat analogues (Sha and Xiong 2020; Shevkani et al. 2015), which probably contributed to the lower consumer acceptance test observed in formulations F3 and F4.

From a nutritional standpoint, formulations F1 and F2 demonstrated promising compositions for the development of high‐quality plant‐based alternatives. The high carbohydrate content (∼40%) observed in these formulations is associated with the inclusion of sorghum, a cereal known for its rich carbohydrate profile (Martinez et al. 2022). Regarding dietary fiber content, formulations F1, F2, and the commercial sample (CO) presented similar concentrations per 100 g serving. Considering the recommendation of 14 g of dietary fiber per 1000 kcal consumed, in a 2000 kcal diet, the formulations F1 and F2 contributed with approximately 46% and 50%, respectively, of the recommended daily fiber intake (Institute of Medicine (IOM) 2005). According to Food and Drug Administration (2023) guidelines, which define a “good source” of fiber as providing 10%–19% of the daily value per serving and an “excellent source” as providing 20% or more, both F1 and F2 can be classified as “excellent sources” of dietary fiber.

Regarding lipid content, the sorghum‐based formulations showed lower levels compared to the commercial burger (CO), due to the reduced addition of soybean oil. Sorghum, in turn, is known for its high concentrations of bound phenolic acids and anthocyanins—compounds that contribute to pH reduction and may promote predominantly non‐covalent phenolic–protein interactions (Shahidi and Dissanayaka 2023). These interactions have the potential to positively influence fat‐related technological properties, such as WHC, gelation, and emulsion stability—key features for maintaining product structure (Jung et al. 2024).

Regarding protein content, burgers F1 and F2 contained approximately 19% of protein, which is lower than the commercial burger (CO), which presented 37.5%. This difference is attributed to the base ingredients used in the formulations—CO being primarily composed of legumes soy and pea, whereas F1 and F2 were mostly made from sorghum cereal and supplemented with cowpea protein isolate (v/v 3.5:1). Nevertheless, despite the lower protein percentage compared to CO, a 100 g serving of the formulations provides 34% of the recommended daily protein intake (Institute of Medicine (IOM) 2005), qualifying them—according to Food and Drug Administration (2023) criteria—as “excellent sources” of protein. Moreover, considering the economic aspect, average producer prices between 2020 and 2023 ranged from $0.34 to $0.52/kg for soybean (the United States) and from $0.38 to $0.45/kg for pea (Canada), whereas sorghum showed lower values, ranging from $0.19 to $0.41/kg (Food and Agriculture Organization of the United Nations (FAO) 2024). This difference reinforces the potential of sorghum as an alternative to reduce the final cost of plant‐based products without compromising their nutritional quality.

For mineral content, formulations F1 and F2 stood out for their higher iron levels compared to CO. The presence of iron in these formulations can be mainly attributed to the cowpea protein isolate, which is recognized as a good source of this mineral, with reported contents ranging from 5.85 to 6.90 mg/100 g (Coelho et al. 2021; Gomes et al. 2022). On the basis of daily intake recommendations for adults, F1 and F2 supplied approximately 69% and 66% of the recommended daily iron intake, respectively (Institute of Medicine (IOM) 2005). For zinc, F1 and F2 contributed with approximately 20% of the daily recommendation, qualifying both formulations as “excellent sources” of these minerals according to Food and Drug Administration (2023) standards.

Regarding antioxidant capacity, formulation F2 stood out with the highest antioxidant potential, which can be attributed to its higher concentration of bioactive compounds such as anthocyanins and phenolic acids. It is likely that the presence of tannins in this formulation, when subjected to thermal processing, underwent depolymerization, releasing bound phenolic acids and thereby increasing their bioavailability. In addition, tannins can form stable complexes with anthocyanins and other phenolic compounds, providing higher protection against thermal degradation (De Morais Cardoso et al. 2017). Accordingly, the formulation F2, prepared with tannin‐sorghum, showed not only a higher profile of phenolic acids, released during processing, but also a higher retention of anthocyanins, compared to formulation F1, based on tannin‐free sorghum. Although the presence of tannins was identified in F2, their content was relatively low, compared to the CMSXS319 sorghum grain (65.5 mg CE/g), possibly due to degradation or complexation during thermal processing. Similar results were reported by Teles et al. (2025), who observed a marked reduction or even the absence of detectable tannins in sorghum flours subjected to cooking processes. These combined factors may have contributed to the superior antioxidant activity observed in F2.

For WHC, no significant differences were observed among the samples, and sorghum is recognized for its ability to retain water (Khoddami et al. 2023). In contrast, the higher OHC observed in the commercial sample (CO) indicates a greater affinity of the soy‐based matrix for oil compared to the sorghum‐based formulations F1 and F2. According to Du et al. (2025), this higher retention can be explained by the emulsifying capacity of soy proteins, which promote the incorporation and stabilization of oil droplets within the protein matrix.

In the instrumental color evaluation, formulations F1 and F2 showed lower L* values compared to CO, indicating a darker appearance. This characteristic can be attributed to the presence of phenolic compounds and anthocyanins—pigments responsible for blue, purple, and red hues in fruits and vegetables—whose color expression is pH‐dependent. It is likely that anthocyanins reduced the pH of the formulations, thereby diminishing lightness and resulting in a darker tone that brings the appearance of the burgers closer to that of traditional meat (Silva et al. 2023). In contrast, the higher L* value observed in the CO sample may be explained by its soy‐based composition, which contains fewer dark pigments and therefore results in a lighter appearance (Ahmad et al. 2022).

For the a* parameter, formulation F1 exhibited the most intense red hue, attributed to the natural reddish color of the BRS 310 sorghum variety, which may positively influence visual acceptance of plant‐based meat analogues. Regarding the b* parameter, associated with yellow tones, the sorghum‐based formulations showed lower values, likely due to their higher anthocyanin content. CO presented the highest b* value, which may be linked to the naturally yellowish color of soy protein.

In terms of chroma index (C*), which reflects color saturation, the sorghum‐based formulations had lower values than CO, also likely due to the genotype's color profile. Finally, with respect to the hue angle (*h*°), F1 and F2 showed values indicative of dark brown tones—a trait considered advantageous for products aiming to replicate the appearance of traditional meat, contributing to a more authentic visual appeal of the developed analogues (Malav et al. 2015; Singh and Sit 2022).

With respect to technological properties, formulation F1 showed higher cooking yield and lower cooking loss compared to the other formulations. On the other hand, both moisture retention and aW were lower in F1 and F2 than in the commercial sample (CO). This behavior can be attributed not only to the type of protein used but also to the presence of phenolic compounds. Although soy proteins—predominant in CO—are well known for their high solubility and gel‐forming capacity, which promote the retention of free water (Sha and Xiong 2020), sorghum and cowpea proteins, when combined with phenolic compounds such as anthocyanins and phenolic acids, tend to form denser and more rigid matrices with a higher proportion of bound water.

Studies have shown that anthocyanins and phenolic compounds can bind to proteins through hydrophobic interactions and hydrogen bonding, forming low‐solubility complexes that reduce water mobility and influence textural properties (Zhang et al. 2024). Although lower aW may reduce the perception of juiciness, it contributes positively to the structural and microbiological stability of the product. Nevertheless, no significant differences were observed in the final diameter among the sorghum‐based formulations and CO, indicating good dimensional stability during thermal processing.

Although F2 received lower sensory scores for the texture attribute—associated with higher instrumental hardness and requiring greater breaking force—this resulted in an inverse correlation between hardness, cohesiveness, and perceived texture (TPA). Plant‐based products often exhibit inferior textural properties compared to meat products, particularly in terms of hardness, cohesiveness, resilience, and chewiness (Souppez et al. 2025). However, the firmness observed in F2 may represent a desirable trait for meat analogues, bringing the texture profile closer to that expected in conventional meat products.

Chewiness showed a direct correlation with gumminess, indicating that samples like CO and F2 required higher masticatory effort. Conversely, F2 also presented a high peak force that was inversely correlated with texture perception, reinforcing the notion that excessive mechanical resistance can negatively impact consumer acceptance. According to Michel et al. (2021), meat alternatives tend to have high acceptance when they successfully mimic the flavor and texture of conventional meat.

The direct correlation between peak force, hardness, and bioactive compounds in F2 suggests that the increased mechanical resistance in this formulation may be attributed to the concentration of anthocyanins and phenolic acids. During processing, the disruption of cellular integrity allows phenolic compounds—originally confined within vacuoles—to interact with proteins and other macromolecules in the food matrix (Günal‐Köroğlu et al. 2023), forming both covalent and non‐covalent bonds that enhance structural rigidity (Schefer et al. 2021).

In summary, the test formulations demonstrated promising nutritional, technological, and sensory properties for meat analogue applications. Despite the greater firmness observed in F2, the results highlight the potential of these ingredients in developing high value‐added plant‐based products. Future research should focus on evaluating the stability of sensory attributes during storage, particularly among vegetarian consumer groups. It is important to note that the commercial sample (CO) contains pea and soy proteins, which are widely used in plant‐based products but present limitations: Pea protein is largely imported, and soybean is recognized as a highly allergenic ingredient, factors that may reduce consumer acceptance. In contrast, sorghum and cowpea represent a promising alternative for the development of affordable, nutritious, and environmentally sustainable plant‐based meat analogues.

## 5 Conclusion

The formulations developed with sorghum and cowpea protein isolate demonstrated good chemical, sensory, and nutritional characteristics, in addition to promising technological performance for meat analogues. The formulation based on tannin‐sorghum presented better nutritional and functional properties due to the presence of tannins, anthocyanins, and phenolic acids. Beyond these attributes, the use of sorghum in high proportions also represents an economic advantage, due to its low production cost. Furthermore, cowpea and sorghum are less allergenic ingredients and more sustainable and resilient crops, relative to legumes commonly employed in plant‐based products, such as soybean and pea.

## Author Contributions

**Andressa Alvarenga Silva**: conceptualization, methodology, data curation, investigation, formal analysis, writing – original draft, software, visualization. **Valéria Aparecida Vieira Queiroz**: conceptualization, data curation, funding acquisition, investigation, methodology, project administration, resources, supervision, validation, visualization. **Francielle Barbosa Pena**: data curation, methodology, writing – original draft, writing – review and editing. **Cícero Beserra de Menezes**: planting of the sorghum genotypes. **Mária Herminia Ferrari Felisberto**: methodology, writing – original draft, writing – review and editing. **Renata Celi Lopes Toledo**: methodology, writing – original draft, writing – review and editing. **Barbara Pereira da Silva**: writing – review and editing, **Hércia Stampini Duarte Martino**: conceptualization, data curation, funding acquisition, investigation, methodology, project administration, resources, supervision, validation, visualization, writing – original draft, writing – review and editing. All authors have read and agreed to the published version of the manuscript.

## Funding

This research was funded by the Coordination for the Improvement of Higher Educational Personnel (CAPES) Foundation (Ministry of Education, Brazil, Financial code 001); Research Support Foundation of the State of Minas Gerais (Fapemig), Financial code 001, National Council of Technological and Scientific Development (CNPq), no. process 403877/2023‐7; H.S.D. Martino is Research Productivity Fellows (no. 312807/2023‐6); and Embrapa Milho e Sorgo (Sete Lagoas, MG, Brazil) who donated sorghum to this study.

## Conflicts of Interest

The authors declare no conflicts of interest.

## Supporting information




**Supplementary Material**: jfds70953‐sup‐0001‐SuppMat.docx

## References

[jfds70953-bib-0001] Ahmad, M. , S. M. H. Qureshi Akbar , et al. 2022. “Plant‐Based Meat Alternatives: Compositional Analysis, Current Development and Challenges.” Applied Food Research 2, no. 2: 100154. 10.1016/j.afres.2022.100154.

[jfds70953-bib-0002] Andreani, G. , G. Sogari , A. Marti , F. Froldi , H. Dagevos , and D. Martini . 2023. “Plant‐Based Meat Alternatives: Technological, Nutritional, Environmental, Market, and Social Challenges and Opportunities.” Nutrients 15, no. 2: 452. 10.3390/nu15020452. 36678323 PMC9861156

[jfds70953-bib-0003] AOAC International . 2019. Official Methods of Analysis of the AOAC International. AOAC International.

[jfds70953-bib-0004] Aschemann‐Witzel, J. , R. F. Gantriis , P. Fraga , and F. J. A. Perez‐Cueto . 2021. “Plant‐Based Food and Protein Trend From a Business Perspective: Markets, Consumers, and the Challenges and Opportunities in the Future.” Critical Reviews in Food Science and Nutrition 61, no. 18: 3119–3128. 10.1080/10408398.2020.1793730. 32654499

[jfds70953-bib-0005] Awika, J. M. , and L. W. Rooney . 2004. “Sorghum Phytochemicals and Their Potential Impact on human Health.” Phytochemistry 65, no. 9: 1199–1221. 10.1016/j.phytochem.2004.04.001. 15184005

[jfds70953-bib-0007] Batista, L. F. , F. Rocha , M. M. S. Dias , A. C. S. Pires , and M. C. T. R. Vidigal . 2023. “Comfort Plant‐Based Food: What Do Consumers Want?—A Focus Group Study With Different Consumers Group.” International Journal of Gastronomy and Food Science 34: 100810. 10.1016/j.ijgfs.2023.100810.

[jfds70953-bib-0008] Bi, Y. , and P. Wang . 2022. “Exploring Drought‐Responsive Crucial Genes in Sorghum.” Iscience 25, no. 11: 105347. 10.1016/j.isci.2022.105347. 36325072 PMC9619295

[jfds70953-bib-0009] Bohrer, B. M. 2019. “An Investigation of the Formulation and Nutritional Composition of Modern Meat Analogue Products.” Food Science and Human Wellness 8, no. 4: 320–329. 10.1016/j.fshw.2019.11.006.

[jfds70953-bib-0011] Chiremba, C. , J. R. N. Taylor , L. W. Rooney , and T. Beta . 2012. “Phenolic Acid Content of Sorghum and Maize Cultivars Varying in Hardness.” Food Chemistry 134, no. 1: 81–88. 10.1016/j.foodchem.2012.02.067. 22500656

[jfds70953-bib-0012] Coelho, R. C. , R. C. F. Barsotti , H. F. Maltez , C. A. Lopes Júnior , and H. S. Barbosa . 2021. “Expanding Information on the Bioaccessibility and Bioavailability of Iron and Zinc in Biofortified Cowpea Seeds.” Food Chemistry 347: 129027. 10.1016/j.foodchem.2021.129027. 33482485

[jfds70953-bib-0013] Cruz, A. G. , J. A. F. Faria , M. A. R. Pollonio , et al. 2011. “Cheeses With Reduced Sodium Content: Effects on Functionality, Public Health Benefits and Sensory Properties.” Trends in Food Science & Technology 22, no. 6: 276–291. 10.1016/j.tifs.2011.02.003.

[jfds70953-bib-0010] De Morais Cardoso, L. , S. S. Pinheiro , H. S. D. Martino , and H. M. Pinheiro‐Sant'Ana . 2017. “Sorghum (*Sorghum bicolor* L.): Nutrients, Bioactive Compounds, and Potential Impact on Human Health.” Critical Reviews in Food Science and Nutrition 57, no. 2: 372–390. 10.1080/10408398.2014.887057. 25875451

[jfds70953-bib-0014] Du, W. , Y. Yin , X. Zhao , et al. 2025. “Optimizing Plant‐Based Meat Alternatives: Effects of Soy Protein Incorporation Methods and Freeze‐Thaw Processing on Microstructure and Quality.” Food Chemistry 493: 145894. 10.1016/j.foodchem.2025.145894. 40816086

[jfds70953-bib-0015] FAO—Food and Agriculture Organization of the United Nations . 2024. World Food Situation—Cereal Supply and Demand Brief. Food and Agriculture Organization of the United Nations.

[jfds70953-bib-0016] Food and Agriculture Organization of the United Nations (FAO) . 2024. FAOSTAT—Producer Prices. Food and Agriculture Organization of the United Nations.

[jfds70953-bib-0017] Food and Drug Administration . 2023. 21 CFR § 101.54—Nutrient Content Claims for “Good Source,” “High,” “More,” and “High Potency.” FDA.

[jfds70953-bib-0018] Gomes, J. C. 1996. Análise de alimentos. Universidade Federal de Viçosa.

[jfds70953-bib-0019] Gomes, M. J. C. , H. S. D. Martino , N. Kolba , et al. 2022. “Zinc Biofortified Cowpea (*Vigna unguiculata* L. Walp.) Soluble Extracts Modulate Assessed Cecal Bacterial Populations and Gut Morphology In Vivo (*Gallus gallus*).” Frontiers in Bioscience (Landmark Edition) 27: 140. 35638407 10.31083/j.fbl2705140

[jfds70953-bib-0020] Günal‐Köroğlu, D. , J. M. Lorenzo , and E. Capanoglu . 2023. “Plant‐Based Protein‐Phenolic Interactions: Effect on Different Matrices and In Vitro Gastrointestinal Digestion.” Food Research International 173: 113269. 10.1016/j.foodres.2023.113269. 37803589

[jfds70953-bib-0021] Hegde, S. R. , S. Thangalakshmi , and R. Singh . 2023. “A Review of Gluten and Sorghum as a Gluten Free Substitute.” Trends in Horticulture 6, no. 2: 2840. 10.24294/th.v6i2.2840.

[jfds70953-bib-0062] Hollweg, G. , P. C. O. Trindade , B. A. dos Santos , et al. 2024. “Development of Plant‐Based Burgers with Partial Replacement of Texturized Soy Protein by Agaricus Bisporus: Effects on Physicochemical and Sensory Properties.” Foods 13, no. 22: 3583. 10.3390/foods13223583. PMC11593058 39593999

[jfds70953-bib-0022] Horax, R. , N. S. Hettiarachchy , P. Chen , and M. Jalaluddin . 2004. “Functional Properties of Protein Isolate From Cowpea (*Vigna unguiculata* L. Walp.).” Journal of Food Science 69, no. 2: fct119–fct121. 10.1111/j.1365-2621.2004.tb15501.x.

[jfds70953-bib-0023] Institute of Medicine (IOM) . 2005. Dietary Reference Intakes for Energy, Carbohydrate, Fiber, Fat, Fatty Acids, Cholesterol, Protein, and Amino Acids. National Academies Press. 10.1016/s0002-8223(02)90346-9 12449285

[jfds70953-bib-0024] Jung, M. , Y. Lee , S. O. Han , and J. E. Hyeon . 2024. “Advancements in Sustainable Plant‐Based Alternatives: Exploring Proteins, Fats, and Manufacturing Challenges in Alternative Meat Production.” Journal of Microbiology and Biotechnology 34, no. 5: 994–1002. 10.4014/jmb.2312.12049. 38379287 PMC11180908

[jfds70953-bib-0025] Khoddami, A. , V. Messina , K. Vadabalija Venkata , A. Farahnaky , C. L. Blanchard , and T. H. Roberts . 2023. “Sorghum in Foods: Functionality and Potential in Innovative Products.” Critical Reviews in Food Science and Nutrition 63, no. 9: 1170–1186. 10.1080/10408398.2021.1960793. 34357823

[jfds70953-bib-0026] Lawless, H. T. , and H. Heymann . 2010. Sensory Evaluation of Food: Principles and Practices. Springer.

[jfds70953-bib-0027] Leite‐Legatti, A. V. , Â. G. Batista , N. R. V. Dragano , et al. 2012. “Jaboticaba Peel: Antioxidant Compounds, Antiproliferative and Antimutagenic Activities.” Food Research International 49, no. 1: 596–603. 10.1016/j.foodres.2012.07.044.

[jfds70953-bib-0028] Macfie, H. J. , N. Bratchell , K. Greenhoff , and L. V. Vallis . 1989. “Designs to Balance the Effect of Order of Presentation and First‐Order Carry‐Over Effects in Hall Tests.” Journal of Sensory Studies 4, no. 2: 129–148. 10.1111/j.1745-459X.1989.tb00463.x.

[jfds70953-bib-0029] Malav, O. P. , S. Talukder , P. Gokulakrishnan , and S. Chand . 2015. “Meat Analog: A Review.” Critical Reviews in Food Science and Nutrition 55, no. 9: 1241–1245. 10.1080/10408398.2012.689381. 24915320

[jfds70953-bib-0030] Martinez, O. D. M. , M. J. C. Gomes , M. Grancieri , et al. 2022. “Sorghum Flour BRS 305 Hybrid Has the Potential to Modulate the Intestinal Microbiota of Rats Fed With a High‐Fat High‐Fructose Diet.” European Journal of Nutrition 62: 647–657. 10.1007/s00394-022-03018-3. 36181539

[jfds70953-bib-0031] Meilgaard, M. C. , B. T. Carr , and G. V. Civille . 1999. Sensory Evaluation Techniques. CRC Press. 10.1201/9781003040729.

[jfds70953-bib-0032] Melo, A. S. , R. R. Costa , F. V. S. Sá , et al. 2024. “Modulation of Drought‐Induced Stress in Cowpea Genotypes Using Exogenous Salicylic Acid.” Plants 13, no. 5: 634. 10.3390/plants13050634. 38475480 PMC10934789

[jfds70953-bib-0033] Merrill, A. L.‐W. B. K. 1995. Energy Value of Foods: Basis and Derivation. US Department of Agriculture.

[jfds70953-bib-0034] Michel, F. , C. Hartmann , and M. Siegrist . 2021. “Consumers' Associations, Perceptions and Acceptance of Meat and Plant‐Based Meat Alternatives.” Food Quality and Preference 87: 104063. 10.1016/j.foodqual.2020.104063.

[jfds70953-bib-0035] Minim, V. P. R. 2013. Sensory Analysis: Consumer Studies. Editora UFV.

[jfds70953-bib-0036] Ministry of Agriculture, Livestock and Supply/Secretariat of Agricultural Defense . 2022. “Portaria SDA no 724, de 23 de dezembro de 2022”. Diário Oficial da União. Brasília, DF, Brazil: Imprensa Nacional.

[jfds70953-bib-0037] Mwamahonje, A. , Z. Mdindikasi , D. Mchau , et al. 2024. “Advances in Sorghum Improvement for Climate Resilience in the Global Arid and Semi‐Arid Tropics: A Review.” Agronomy 14, no. 12: 3025. 10.3390/agronomy14123025.

[jfds70953-bib-0039] Paes, L. T. , C. T. D. S. D'Almeida , M. A. V. Do Carmo , et al. 2024. “Phenolic‐Rich Extracts From Toasted White and Tannin Sorghum Flours Have Distinct Profiles Influencing Their Antioxidant, Antiproliferative, Anti‐Adhesive, Anti‐Invasive, and Antimalarial Activities.” Food Research International 176: 113739. 10.1016/j.foodres.2023.113739. 38163694

[jfds70953-bib-0040] Pavan, V. , R. A. S. Sancho , and G. M. Pastore . 2014. “The Effect of In Vitro Digestion on the Antioxidant Activity of Fruit Extracts (*Carica papaya*, *Artocarpus heterophyllus* and *Annona marcgravii*).” LWT—Food Science and Technology 59, no. 2: 1247–1251. 10.1016/j.lwt.2014.05.040.

[jfds70953-bib-0041] Peyrano, F. , F. Speroni , and M. V. Avanza . 2016. “Physicochemical and Functional Properties of Cowpea Protein Isolates Treated With Temperature or High Hydrostatic Pressure.” Innovative Food Science and Emerging Technologies 33: 38–46. 10.1016/j.ifset.2015.10.014.

[jfds70953-bib-0042] Pi, X. , Y. Sun , G. Fu , Z. Wu , and J. Cheng . 2021. “Effect of Processing on Soybean Allergens and Their Allergenicity.” Trends in Food Science & Technology 118: 316–327. 10.1016/j.tifs.2021.10.006.

[jfds70953-bib-0043] Price, M. L. , S. Van Scoyoc , and L. G. Butler . 1978. “A Critical Evaluation of the Vanillin Reaction as an Assay for Tannin in Sorghum Grain.” Journal of Agricultural and Food Chemistry 26: 1214–1218.

[jfds70953-bib-0044] Research and Markets . 2025. Meat Analogue Market Report: Trends, Forecast and Competitive Analysis to 2031. Research and Markets. https://www.researchandmarkets.com/reports/6167796/meat‐analogue‐market‐report‐trends‐forecast#src‐pos‐1.

[jfds70953-bib-0045] Rufino, M. D. S. M. , R. E. Alves , E. S. de Brito , et al. 2007. Metodologia Científica: Determinação da Atividade Antioxidante Total em Frutas Pela Captura do Radical Livre ABTS°+. Embrapa Agroindústria Tropical.

[jfds70953-bib-0046] Samard, S. , T.‐T. Maung , B.‐Y. Gu , M.‐H. Kim , and G.‐H. Ryu . 2021. “Influences of Extrusion Parameters on Physicochemical Properties of Textured Vegetable Proteins and Its Meatless Burger Patty.” Food Science and Biotechnology 30, no. 3: 395–403. 10.1007/s10068-021-00879-y. 33868750 PMC8017041

[jfds70953-bib-0047] Schefer, S. , M. Oest , and S. Rohn . 2021. “Interactions Between Phenolic Acids, Proteins, and Carbohydrates—Influence on Dough and Bread Properties.” Foods 10, no. 11: 2798. 10.3390/foods10112798. 34829079 PMC8624349

[jfds70953-bib-0048] Selani, M. M. , G. A. N. Shirado , G. B. Margiotta , et al. 2016. “Effects of Pineapple Byproduct and Canola Oil as Fat Replacers on Physicochemical and Sensory Qualities of Low‐Fat Beef Burger.” Meat Science 112: 69–76. 10.1016/j.meatsci.2015.10.020. 26562792

[jfds70953-bib-0049] Sha, L. , and Y. L. Xiong . 2020. “Plant Protein‐Based Alternatives of Reconstructed Meat: Science, Technology, and Challenges.” Trends in Food Science & Technology 102: 51–61. 10.1016/j.tifs.2020.05.022.

[jfds70953-bib-0050] Shahidi, F. , and C. S. Dissanayaka . 2023. “Phenolic‐Protein Interactions: Insight From In‐Silico Analyses—A Review.” Food Production, Processing and Nutrition 5, no. 1: 2. 10.1186/s43014-022-00121-0.

[jfds70953-bib-0051] Shao, Y. , F. Xu , X. Sun , J. Bao , and T. Beta . 2014. “Phenolic Acids, Anthocyanins, and Antioxidant Capacity in Rice (*Oryza sativa* L.) Grains at Four Stages of Development After Flowering.” Food Chemistry 143: 90–96. 10.1016/j.foodchem.2013.07.042. 24054217

[jfds70953-bib-0052] Shevkani, K. , A. Kaur , S. Kumar , and N. Singh . 2015. “Cowpea Protein Isolates: Functional Properties and Application in Gluten‐Free Rice Muffins.” LWT—Food Science and Technology 63, no. 2: 927–933. 10.1016/j.lwt.2015.04.058.

[jfds70953-bib-0053] Silva, A. , V. Silva , G. Igrejas , et al. 2023. “Phenolic Compounds Classification and Their Distribution in Winemaking By‐Products.” European Food Research and Technology 249, no. 2: 207–239. 10.1007/s00217-022-04163-z.

[jfds70953-bib-0054] Singh, A. , and N. Sit . 2022. “Meat Analogues: Types, Methods of Production and Their Effect on Attributes of Developed Meat Analogues.” Food and Bioprocess Technology 15, no. 12: 2664–2682. 10.1007/s11947-022-02859-4.

[jfds70953-bib-0055] Souppez, J.‐B. R. G. , B. A. S. Dages , G. S. Pavar , J. Fabian , J. M. Thomas , and E. Theodosiou . 2025. “Mechanical Properties and Texture Profile Analysis of Beef Burgers and Plant‐Based Analogues.” Journal of Food Engineering 385: 112259. 10.1016/j.jfoodeng.2024.112259.

[jfds70953-bib-0056] Stone, A. K. , A. Karalash , R. T. Tyler , T. D. Warkentin , and M. T. Nickerson . 2015. “Functional Attributes of Pea Protein Isolates Prepared Using Different Extraction Methods and Cultivars.” Food Research International 76: 31–38. 10.1016/j.foodres.2014.11.017.

[jfds70953-bib-0057] Teles, A. G. , W. A. da Silva , C. B. de Menezes , et al. 2025. “Processes Applied to Whole Sorghum Flour: Effects on Technological, Antioxidant and Sensory Properties of Gluten‐Free Cakes.” Journal of Food Measurement and Characterization 19, no. 8: 5848–5856. 10.1007/s11694-025-03358-8.

[jfds70953-bib-0058] Villanueva, N. D. M. , A. J. Petenate , and M. A. A. P. Da Silva . 2005. “Performance of the Hybrid Hedonic Scale as Compared to the Traditional Hedonic, Self‐Adjusting and Ranking Scales.” Food Quality and Preference 16, no. 8: 691–703. 10.1016/j.foodqual.2005.03.013.

[jfds70953-bib-0060] Yang, L. , J. D. Browning , and J. M. Awika . 2009. “Sorghum 3‐Deoxyanthocyanins Possess Strong Phase II Enzyme Inducer Activity and Cancer Cell Growth Inhibition Properties.” Journal of Agricultural and Food Chemistry 57, no. 5: 1797–1804. 10.1021/jf8035066. 19256554

[jfds70953-bib-0059] Yang, L. , K. F. Allred , B. Geera , C. D. Allred , and J. M. Awika . 2012. “Sorghum Phenolics Demonstrate Estrogenic Action and Induce Apoptosis in Nonmalignant Colonocytes.” Nutrition and Cancer 64, no. 3: 419–427. 10.1080/01635581.2012.657333. 22369068

[jfds70953-bib-0061] Zhang, J. , B. Hong , M. Abdollahi , H. Wu , and I. Undeland . 2024. “Role of Lingonberry Press Cake in Producing Stable Herring Protein Isolates via pH‐Shift Processing: A Dose Response Study.” Food Chemistry: X 22: 101456. 10.1016/j.fochx.2024.101456. 38808166 PMC11130683

