# Supplementary material for: The Incorporation of Sorghum and Cowpea Protein Isolate Into Plant‐Based Burgers Improved Their Physicochemical and Sensory Properties
Source: J Food Sci. 2026 Mar 22;91(3):e70953. doi: 10.1111/1750-3841.70953 (PMC13006479; doi:10.1111/1750-3841.70953)
Supplement: Supplementary file 1 — Supplementary Material: jfds70953‐sup‐0001‐SuppMat.docx [file JFDS-91-0-s001.docx]

##
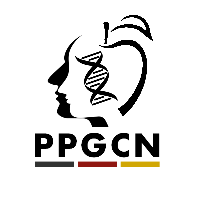


Universidade Federal de Viçosa

Centro de Ciências Biológicas e da Saúde

Programa de Pós Graduação em Ciência da Nutrição

Av. P.H. Rolfs S/N - Campus Universitário. Viçosa, MG. – CEP.: 36570.000 Tel.: 031 3612-5186 Fax: 031 3612-5187

**INFORMED CONSENT FORM**

You are being invited to participate as a volunteer in a research study **aimed at developing plant-based burgers** formulated with sorghum and cowpea protein isolate.

You will attend a single session at the Sensory Analysis and New Product Processing Laboratory of the Department of Nutrition and Health (DNS) at the Federal University of Viçosa (UFV), where you will consume different formulations of plant-based burgers. A sensory evaluation will be conducted to assess flavor, color, aroma, texture, and overall impression for each formulation. Mineral water will be provided for palate cleansing between the evaluation of each sample.

Along with the samples, you will receive an evaluation form in which you will be asked to rate the products based on flavor, texture, appearance, and overall impression. A 9-point hedonic scale will be used, with a score of 9 corresponding to “like extremely” and a score of 1 corresponding to “dislike extremely”. In addition, purchase intention will be evaluated using a 5-point hedonic scale, with a score of 1 corresponding to “I would definitely buy it” and a score of 5 corresponding to “I would definitely not buy it.”

The data collected will be stored in a manner that ensures the privacy and confidentiality of the participants and will be accessible only to the research team involved in the project. These data may be used for the publication of scientific papers and other academic materials, with the anonymity of the volunteers fully guaranteed.

**Allergy information:** The burgers do not contain gluten. You declare that you are aware of this information; however, in the event of any discomfort, the research team will be responsible for providing appropriate care to the volunteers.

Participation in this study will not involve any costs to you, nor will you receive any financial compensation or benefit. You will receive full and immediate assistance for as long as necessary, with all related costs being entirely the responsibility of the researchers. The research team will not be responsible for health conditions resulting from information not disclosed by the participant that may interfere with their health status.

This study will not result in any financial loss to the participant, and all necessary materials will be provided by the research team. There will be no employment relationship or any form of remuneration between the volunteer and the researchers.

You are guaranteed full freedom to refuse to participate or to withdraw your consent at any stage of the research, without the need for prior notification. Your participation is voluntary, and refusal to participate will not result in any penalty or change in the manner in which you are treated by the researcher. You will not be identified in any publication resulting from this study. Your name or any material that could identify your participation will not be disclosed without your permission.

This informed consent form is printed in two original copies, one of which will be retained by the responsible researcher and the other provided to you. All pages must be initialed by you and by the responsible researcher. The questionnaires collected in this study will be stored for analyses related to this project and may also be used in future research within the same field of study.

The results of this project and of future related studies may be presented, communicated, and/or published in the scientific literature, with your confidentiality and privacy always preserved. The data and research instruments used in this study will be stored by the responsible researcher for a period of **five (5) years** after the conclusion of the research and will be destroyed thereafter.

The researchers will treat your identity in accordance with professional standards of confidentiality and privacy, in compliance with Brazilian legislation, particularly **Resolution No. 466/2012 of the Brazilian National Health Council**, and the information collected will be used exclusively for academic and scientific purposes.

I, _____________________________________________________________________________, identification document ________________________, contact information _________________________________________________, have been clearly and thoroughly informed about the objectives of this research study and have had my questions adequately answered. I understand that I may request additional information at any time and may change my decision to participate if I so wish. I hereby declare that I agree to participate in this study. I have received one original copy of this informed consent form and was given the opportunity to read it and clarify any questions.

Viçosa, Brazil, ______, ______________, 2025.

| Participant’s Signature | **Prof. Hércia Stampini Duarte Martino** **Principal Investigator (DNS/UFV)** |
| --- | --- |

| Andressa Alvarenga Silva  PhD Candidate (DNS/UFV) |
| --- |

**Names and contact information of the investigators:**

Principal Investigator: Prof. Hércia Stampini Duarte Martino
Address: Federal University of Viçosa – Department of Food Technology
University Campus – ZIP Code: 36571-000, Viçosa, MG, Brazil
Phone: +55 31 3612-5207
E-mail: hercia@ufv.br

In case of disagreement or concerns regarding ethical aspects of this research, you may contact:

CEP/UFV – Human Research Ethics Committee
Federal University of Viçosa – Arthur Bernardes Building, lower floor
Av. PH Rolfs, s/n – University Campus – ZIP Code: 36570-900, Viçosa, MG, Brazil
Phone: +55 31 3612-2316
E-mail: cep@ufv.br
Website: [www.cep.ufv.br](http://www.cep.ufv.br)
Office hours: Monday to Friday, from 8:00 a.m. to 12:00 p.m.
